# Quantitative Analysis of Monocyte Subpopulations in Murine Atherosclerotic Plaques by Multiphoton Microscopy

**DOI:** 10.1371/journal.pone.0044823

**Published:** 2012-09-14

**Authors:** Abigail S. Haka, Stephane Potteaux, Haley Fraser, Gwendalyn J. Randolph, Frederick R. Maxfield

**Affiliations:** 1 Department of Biochemistry, Weill Cornell Medical College, New York, New York, United States of America; 2 Department of Gene and Cell Medicine and the Immunology Institute, Mount Sinai School of Medicine, New York, New York, United States of America; Tufts University, United States of America

## Abstract

The progressive accumulation of monocyte-derived cells in the atherosclerotic plaque is a hallmark of atherosclerosis. However, it is now appreciated that monocytes represent a heterogeneous circulating population of cells that differ in functionality. New approaches are needed to investigate the role of monocyte subpopulations in atherosclerosis since a detailed understanding of their differential mobilization, recruitment, survival and emigration during atherogenesis is of particular importance for development of successful therapeutic strategies. We present a novel methodology for the *in vivo* examination of monocyte subpopulations in mouse models of atherosclerosis. This approach combines cellular labeling by fluorescent beads with multiphoton microscopy to visualize and monitor monocyte subpopulations in living animals. First, we show that multiphoton microscopy is an accurate and timesaving technique to analyze monocyte subpopulation trafficking and localization in plaques in excised tissues. Next, we demonstrate that multiphoton microscopy can be used to monitor monocyte subpopulation trafficking in atherosclerotic plaques in living animals. This novel methodology should have broad applications and facilitate new insights into the pathogenesis of atherosclerosis and other inflammatory diseases.

## Introduction

Atherosclerosis is the leading cause of death in developed countries [1]. It is a chronic inflammatory disease of the arterial wall, which begins with local disrupted shear stress and passive accumulation of modified, aggregated lipoproteins [2]. Further manifestation of the disease is the result of unbalanced trafficking of monocytes in and out of the atherosclerotic blood vessel. As monocytes infiltrate the intima, they fail to emigrate with their lipid loads, and consequently, they contribute to the development of plaques [3], [4]. Two subpopulations of monocytes, referred to as classical (Gr1^hi^) and non-classical (Gr1^lo^) monocytes, have been described based on their differential antigen marker expression, such as chemokine receptors [5]. In the mouse, classical monocytes promote inflammation, expand in hypercholesterolemic conditions and give rise to macrophages within the plaque [6]. Non-classical monocytes attenuate inflammation and are predisposed to becoming CD11c+ dendritic-like cells within lesions [6]. Although both subpopulations enter the plaque and participate in plaque development, little is known regarding their specific migratory behaviors and roles in atherosclerosis. New approaches are needed to investigate the role of monocyte subpopulations in atherosclerosis. In particular, the ability to image monocyte subpopulation behavior in plaques in living animals would significantly enhance our understanding of atherosclerosis pathophysiology.

Few techniques are currently available to trace monocyte subpopulations *in vivo* in mice [7]. The Randolph lab recently developed a quantitative technique that monitors monocyte subpopulation trafficking in the atherosclerotic plaque [4], [6], [8]. This approach uses non-degradable fluorescent latex microbeads as permanent tracers to pulse-label and follow circulating monocytes that subsequently enter the plaque. Counting the beads in the plaque at different time points or under various treatment conditions allows quantification of both monocyte subpopulation recruitment [6] and emigration in atherosclerosis [4]. It has been accomplished by excising, fixing and sectioning the vessel, and manually counting the number of beads in each section with a fluorescence microscope. Although the approach is used by several laboratories and has provided novel insights into the role of monocyte subpopulations in atherosclerosis [9], [10], [11], the tissue sectioning and manual bead quantification are laborious and time consuming. Further, to date most of our knowledge on the activity of monocyte populations has been obtained with *ex vivo* readouts (*e.g.,* histology, flow cytometry). While such experiments are useful, immune processes are dynamic and thus are best studied in real time in living animals. Consequently, the use of an alternative method that allows the examination and quantification of bead-labeled monocytes in atherosclerotic plaques, in living animals is very attractive.

We present a novel methodology for the *in vivo* examination of monocyte subpopulations in mouse models of atherosclerosis. There were two major goals in the development of this approach. The first was to implement an improved method for quantifying bead-labeled cells in excised tissues. This was achieved through the use of optical rather than mechanical tissue sectioning and automated data collection and analysis. The second was to extend the approach to intravital examination of monocyte trafficking, a realm not attainable with conventional methodologies. To meet these goals, we combined the monocyte subpopulation bead-labeling approach with multiphoton microscopy to tag and monitor monocyte subpopulations in living animals.

Multiphoton microscopy is a promising optical technique for imaging the spatiotemporal trafficking of cells in thick tissues and living animals [12], [13]. Similar to conventional histology, multiphoton imaging enables direct visualization of tissue morphology and thus can predict disease state [14]. However, unlike histology, multiphoton imaging does not require tissue processing or the use of exogenous dyes. Several plaque components can be imaged using two-photon fluorescence and second-harmonic generation microscopic techniques [15], [16], [17]. In two-photon excitation, two photons of excitation light are ‘simultaneously’ absorbed for each excitation event and traditional fluorescence emission is generated. Thus, endogenous tissue autofluorescence, which arises from cytoplasmic flavins, elastin, neutral lipids and calcifications, can readily be visualized in three dimensions [16], [18]. Second-harmonic generation is a nonlinear process in which two photons interacting with a nonlinear material are ‘simultaneously’ scattered to form a single photon with twice the energy/frequency and half the wavelength of the excitation photons [19]. Fibrillar collagen is an important structural component of atherosclerotic lesions, and it is an extremely bright second-harmonic generator [18]. The ability to visualize tissue architecture allows multiphoton images of unprocessed tissue to be used to localize monocytes in different anatomical regions of the plaque and to provide information on disease progression. Further, although there is a wealth of information available from endogenous fluorophores and light scatterers, exogenous dyes can also be employed to indicate particular features of interest.

Multiphoton microscopy has several advantages over conventional fluorescence microscopy for tissue imaging [20]. It relies on infrared excitation light, which confers increased penetration depths due to minimal tissue scattering/absorption of the excitation beam. This allows investigation of thick tissue specimens. Two-photon excitation occurs only at the focal point of the microscope rather than through the entire excitation beam profile, as in conventional fluorescence microscopy. The benefit of localized excitation is that emission is restricted to the narrow focal region, providing sectioning ability without the use of a pinhole in the emission lightpath. This permits optical sectioning of thick tissue specimens. Additionally, the highly localized excitation minimizes sample photobleaching, thereby reducing tissue photodamage. Further, light in the infrared region reduces the risk of tissue damage that occurs at visible and ultraviolet wavelengths. The deep penetration depth and the reduced photobleaching and tissue damage make multiphoton microscopy well suited for the *in vivo* examination of cell behavior.

Although multiphoton microscopy is amenable to intravital imaging, it is usually limited to biological tissues that can be isolated from the anesthetized mouse or easily stabilized [21], [22], [23], [24]. A handful of pilot studies have demonstrated the ability to obtain intravital images of atherosclerotic plaques using multiphoton microscopy. However, the approach is very challenging and has not yet permitted cell trafficking in plaques to be visualized [15], [25], [26]. Obstacles to intravital imaging of atherosclerosis include physical restrictions of the microscope objective, the accessibility of atherosclerotic vessels and movements associated with the heartbeat, breathing and other animal motions.

In this study, we use multiphoton microscopy as a new tool to study monocyte subpopulations in atherosclerotic plaques. First, we show that multiphoton microscopy is an accurate and timesaving technique to analyze monocyte subpopulation trafficking and localization in plaques in excised tissues. Results obtained via sectioning and manual bead counting are compared to those obtained with multiphoton microscopy and automated image analysis. Multiphoton images of bead-positive monocyte-derived cells localized in various structural components of the plaque are also presented. Next, for the first time we show that multiphoton microscopy can be used to monitor monocyte subpopulation trafficking in the plaque in live animals. We acquired intravital time-lapse multiphoton images of non-classical monocyte adhesion to an atherosclerotic plaque in the abdominal aorta of an ApoE^−/−^ mouse. Intravital multiphoton imaging of bead-positive Gr1^lo^ monocyte accumulation in the plaque and interaction with fluorescently labeled low density lipoprotein (LDL) is also presented. We demonstrate that multiphoton microscopy is a promising tool to examine the role of monocyte subpopulations in atherosclerosis.

## Methods

### Animals

#### Ethics statement

Mice were housed in a pathogen-free environment at Mount Sinai School of Medicine or Weill Cornell Medical College and used in accordance with protocols approved by the Institutional Animal Care and Utilization Committee at each institution.

#### Day 1 vs. day 5 experiment

10 ApoE^−/−^ female mice were purchased from Jackson laboratories. They were transitioned at 5 weeks of age to a high-fat diet (21% milk fat, 0.15% cholesterol; Harlan Teklad) and maintained on the diet for 23 weeks in order to develop large lesions in the lesser curvature of the aorta. Mice were separated in 2 groups and sacrificed either 1 day or 5 days after Gr1^lo^ labeling.

#### Simvastatin experiment

6 ApoE^−/−^ male retired breeders were purchased from Jackson laboratories. After 9 months on chow diet, 3 males were treated daily with simvastatin (intraperitoneal injection of 0.57 mg/kg/day, Zocor, Merck, Whitehouse Station, NJ) for 10 days. As a control, 3 mice were treated with the vehicle containing phosphate buffered saline (PBS). 5 days after the treatment had begun, Gr1^lo^ monocytes were labeled *in vivo* and allowed to accumulate in the plaque for the last 5 days of treatment. Mice were then euthanized via carbon dioxide inhalation.

#### Intravital imaging

10 ApoE^−/−^ female mice were purchased from Jackson laboratories and placed on a high-fat diet for one year. Monocyte recruitment in plaques was imaged 2 hrs after bead injection. Monocyte-derived cell accumulation in the plaque was monitored 24 hrs after bead injection. After imaging, animals were euthanized by carbon dioxide inhalation.

### Monocyte Labeling

1-µm Fluoresbrite fluorescein isothiocyanate (FITC)-dyed (YG) plain microspheres (Polysciences, Inc., Warrington, PA) were diluted 1∶4 in sterile PBS, and 250 µl of the solution was intravenously injected in order to selectively label Gr1^lo^ monocytes [4]. The labeling efficiency of each mouse was verified by flow cytometry one day after bead injection. 100 µl of blood was drawn by submandibular puncture. After red blood cell lysis, monocytes were identified by flow cytometry, using fluorochrome-conjugated combinations of the following mAbs: CD115 (PE conjugated anti-mouse CD115, clone AFS98; eBioscience, San Diego, CA), CD45 (APC-Cy7 anti-mouse CD45, clone 30-F11; BioLegend, San Diego, CA) and Gr-1 (PerCP/Cy5.5 anti-mouse Ly-6G/Ly-6C (Gr1); BioLegend).

### Tissue Preparation and Staining

After euthanasia, aortas were flushed with 10 ml of 2 mM ethylenediaminetetraacetic acid (EDTA) in PBS, excised and fixed with 4% paraformaldehyde. Opening the aorta *en face* revealed the lesser curvature. The brachiocephalic artery remained intact. Plaques were visualized with oil red O (ORO) (Sigma Aldrich St. Louis, MO) 5% coloration. Hearts were fixed with 4% paraformaldehyde, frozen in Tissue Tek and sectioned (8 µm steps). Macrophages in plaques were defined as CD68^+^ and DAPI^+^ (100 ng/ml) cells. CD68 staining was done using a purified rat anti-mouse CD68 (cat# MCA 1957, clone FA-11, dilution 1/100, Serotec, Raleigh, NC) and revealed by incubation with a secondary antibody anti-rat Cy3 IgG (cat# 712-165-150, dilution 1/300, Jackson ImmunoResearch West Grove, PA). Images were acquired with a 20x/0.5 numerical aperture (NA), dry objective (Leica DM RA2, Bannockburn, IL).

### Instrumentation

Images were acquired using a custom-built multiphoton microscope. Excitation is provided by a femtosecond pulsed Ti-Sapphire laser (Mai Tai HP, Spectra-Physics, Mountain View, CA), which is tunable from 700 to 1020 nm. In this study, 850 nm excitation was used. However, excitation can be tuned to enhance particular features of interest. Laser power is modulated with a Pockels cell (Conoptics Inc., Danbury, CT). Power levels between 60 and 80 mW were employed. The microscope is an upright Olympus BX61WI (Olympus America, Inc., Center Valley, PA). A BioRad 1024 scan head is used for raster scanning the sample, and the system is run using the BioRad LaserSharp 2000 image acquisition software.

Signal is collected in three detection channels, using Hamamatsu bialkali photomultiplier tubes. The filters used in this study acquire fluorescence and second harmonic generation signals from 380 to 440 nm (collagen second harmonic generation), 490 to 530 nm (beads, collagen and elastin autofluorescence) and 530 to 650 nm (neutral lipids stained with ORO). Excitation light was focused and collected with a 10x/0.4 NA, dry objective (Olympus America, Inc., Center Valley, PA). A cooled charge coupled device camera is attached to the microscope, so widefield white light and fluorescence images of the specimen can also be recorded. Thick samples are illuminated with white light from an external gooseneck light source. A custom-written xyz image tiling software was used to create composite images of the entire lesser curvature of the murine aorta. Similar tiling software is available on most commercially available instruments. 10% overlap was employed between adjacent z-stacks. The total area covered ranged from 9 to 20 mm^2^ depending on lesion size (determined by the area of positive ORO staining). A zoom of 5, axial step size of 10 µm and scan rate of 166 lines per second were employed for tiling image acquisition. The optical inhomogeneity of biological tissues compromises resolution [27]. Thus, we relied on experimental data to optimize our imaging conditions with the goal of covering the largest area in the shortest amount of time without missing any beads. With an axial step size of 10 µm, a single bead could be seen in at least two sections.

### Intravital Imaging

Fluorescent beads were injected to label circulating monocytes 24 hrs or 2 hrs prior to microscopic imaging. In some experiments, we also injected fluorescent LDL 24 hrs prior to imaging in order to visualize the lipid-rich areas of the plaque. We isolated LDL from fresh human plasma by preparative ultracentrifugation as described previously [28]. LDL was fluorescently labeled with AlexaFluor546 (Alexa546) purchased from Invitrogen (Carlsbad, CA). One day prior to the acquisition of multiphoton data, 250 µl (1 mg/ml) of Alexa546-labeled LDL was injected into the lateral tail vein.

Animals were anesthetized via inhalation of isofluorane (VetEquip, Pleasanton, CA) and wrapped in a water-circulating warm blanket to maintain body temperature at 37°C. After assuring adequate anesthesia, the abdominal aorta was exposed by opening the peritoneal cavity and gently pushing organs to the side. The mouse was then placed on a custom built stage insert and transferred to the microscope. Inhalation anesthesia was delivered for the duration of the experiment. All intravital images were acquired using a 10x/0.4 NA objective. The Ti-sapphire laser was tuned to 800 nm excitation for all intravital imaging except Alexa546-LDL imaging, which was performed at 850 nm excitation. The power of the laser was 60 mW on the sample, and an imaging speed of 488 lines per second covering an area of 128×128 pixels was employed. This resulted in acquisition of about 4 frames per second.

### Data Analysis

All data were analyzed with MetaMorph image analysis software (Molecular Devices Corporation, Downington, PA). Bead quantification was preformed using the count nuclei command with settings of a minimum diameter of 0.5 microns and a maximum diameter of 1.5 microns. The values for the minimum and maximum bead diameters were chosen based on the observed size of the fluorescent bead images in the tissue. A threshold value for bead brightness above background was varied for two representative data sets (one composite image from day 1 and one composite image from day 5 following monocyte subpopulation labeling) until the agreement between the number of beads determined by the algorithm and by visual inspection was optimized. Using this threshold, automated quantification was performed on a sum projection of each individual z-stack acquired in the 490 to 530 nm channel. Because the data were acquired with 10% overlap between adjacent z-stacks, code was written to prevent repeated counting of individual beads within this overlap. Composite image stitching and three-dimensional reconstructions were also performed using MetaMorph. Statistical analysis was performed using a one tailed Student’s t-test. To correct for the axial distortions in the three-dimensional reconstruction of the brachiocephalic artery, beads in each image plane were identified using the count nuclei command with settings of a minimum diameter of 0.5 microns and a maximum diameter of 1.5 microns. A higher threshold value was chosen for the difference between the bead fluorescence signal and the local background so that only beads in the plane of best focus, where their signal was brightest, were identified by the algorithm. Processed images of the beads identified in this manner could be used to more precisely identify the position of the fluorescent beads in the tissue.

**Figure 1:**
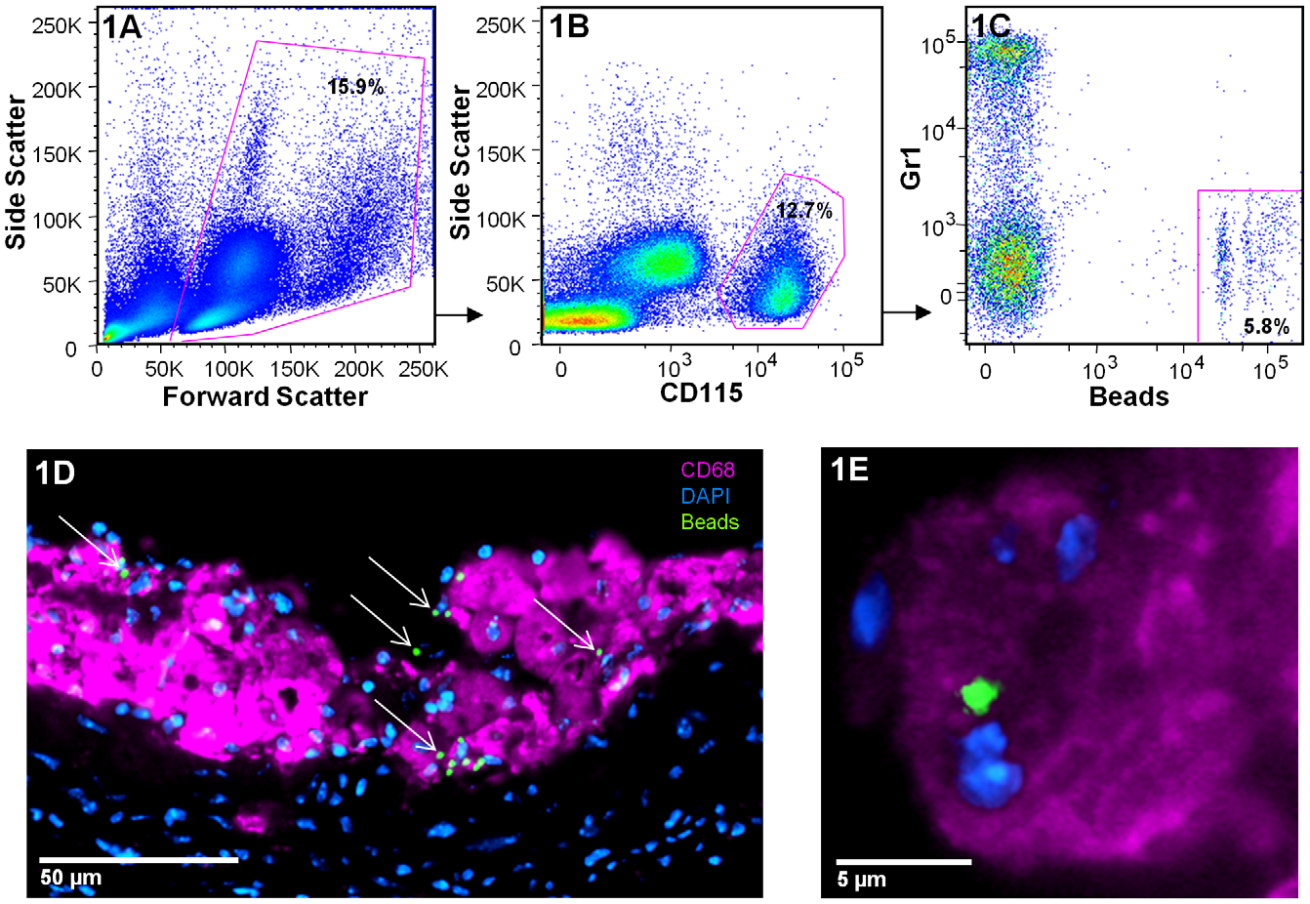
Verification of monocyte subpopulation bead-labeling. A –**C)** Flow cytometry gating strategy used to calculate the monocyte subpopulation bead-labeling efficiency in the blood. After gating on live cells (A), monocytes are detected by their high expression of CD115 (B). In this representative plot, 12.7% of leukocytes are monocytes. Monocytes can then be separated in 2 major populations based on their expression of Gr1 (C). In this example, 5.8% of total monocytes are bead-positive Gr1^lo^. Specific labeling of monocytes can also be visualized in plaques via sectioning of the tissue and immunofluorescence. **D)** A representative image of an atherosclerotic plaque after sectioning of the aortic sinus acquired 5 days following monocyte subpopulation labeling. Bead-positive cells are indicated by arrows. **E)** Beads can be seen to associate with CD68+ macrophages.

## Results

### Multiphoton Microscopy for the Quantification of Monocyte Trafficking in Atherosclerotic Plaques

The monocyte subpopulation labeling efficiency for each mouse was verified by flow cytometry prior to multiphoton data acquisition [8]. After gating on circulating live cells (Figure 1A), monocytes were detected by their high expression of CD115 (Figure 1B). In this representative plot, 12.7% of leukocytes are monocytes. Monocytes can then be separated into two major populations based on their expression of Gr1. In this example, the bead injection specifically labeled non-classical monocytes, and 5.8% of the total blood monocytes are bead-positive Gr1^lo^. Very few beads are found in Gr1^hi^ cells (Figure 1C). Specific labeling of monocytes can also be visualized in plaques via sectioning of the aortic sinus and immunofluorescence. As an example, Figure 1D shows a representative image of monocyte-derived cells (indicated by arrows) in the plaque after staining for CD68. In this image the tissue was harvested 5 days after monocyte subpopulation labeling. It is apparent that bead-labeled cells are associated with CD68-labeled macrophages (Figure 1E). In an earlier study, CD68 distribution was compared with F4/80 and Moma-2 staining [4]. Results were identical for all markers examined indicating that the bead-labeled cells are macrophages. Following confirmation of the success of subpopulation labeling, monocyte trafficking using multiphoton microscopy can be examined in the tissues.

**Figure 2:**
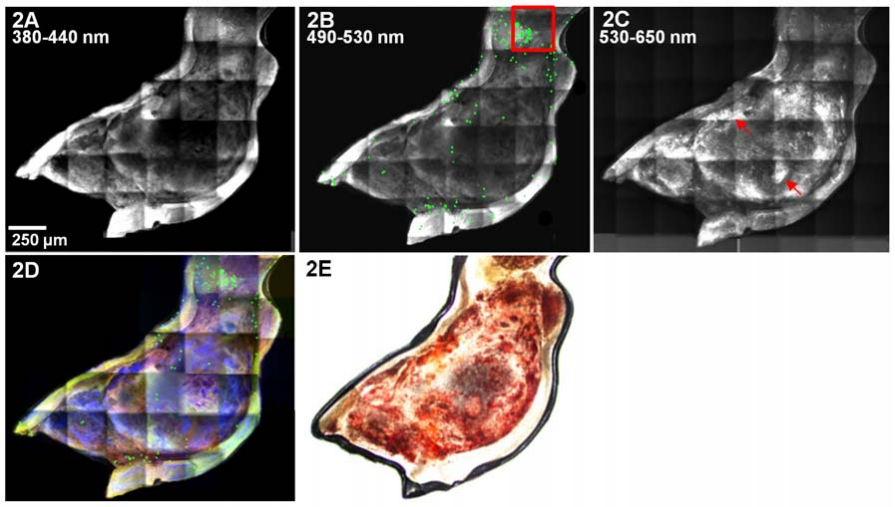
Composite multiphoton images of an atherosclerotic plaque in the lesser curvature of an ApoE−/− mouse. Composite images were generated for each mouse and used to quantify the number of bead-positive monocytes in the plaque. In this example, data were generated from tiling of 28 individual z-stacks and collected from **A)** 380 to 440 nm (second harmonic generation - mainly from collagen), **B)** 490 to 530 nm (bead fluorescence taken into the plaque in monocyte-derived cells; autofluorescence from collagen and elastin. Beads are dilated to 20 µm to facilitate visualization. Adherent bead-labeled monocytes show a specific distribution in the lesional regions, with a preferential accumulation at the edge of the plaque where shear stress is typically lower, red box) and **C)** 530 to 650 nm (ORO fluorescence in neutral lipid deposits). **D)** Overlay composite image. Signal collected from 380 to 440 nm is shown in red, 490 to 530 nm in green, and 530 to 650 nm in blue. **E)** A white light image of the aorta shown in A–D. The tissue was stained with ORO to visually identify the area for multiphoton data collection, so the plaque appears red.

To validate the accuracy of multiphoton microscopy for the quantification of monocyte subpopulation trafficking in atherosclerosis, results obtained via sectioning and manual bead counting were compared to those obtained with multiphoton microscopy and automated image analysis. We performed two independent experiments, one identical in design to a previous experiment [6] and one new experiment, in which monocyte accumulation in plaques was measured. In the first experiment, we determined the number of bead-positive monocytes that infiltrated the plaques of ApoE^−/−^ mice 1 day or 5 days after labeling. It has been shown that monocytes progressively accumulate in the plaque over time [6], thus we expected a significant difference in the number of bead-positive monocytes that infiltrate the plaque 1 day and 5 days after labeling. In the second experiment, non-classical monocyte recruitment to the plaque was quantified in mice given simvastatin or vehicle control. Simvastatin is an HMG CoA reductase inhibitor that reduces cholesterol synthesis and secretion of lipoproteins from the liver [29], and it is a known modulator of monocyte trafficking [30]. To quantify the number of bead-positive monocytes in each plaque, multiphoton tiling of the lesser curvature of the aorta was performed, and large composite images of each sample were generated.

**Figure 3:**
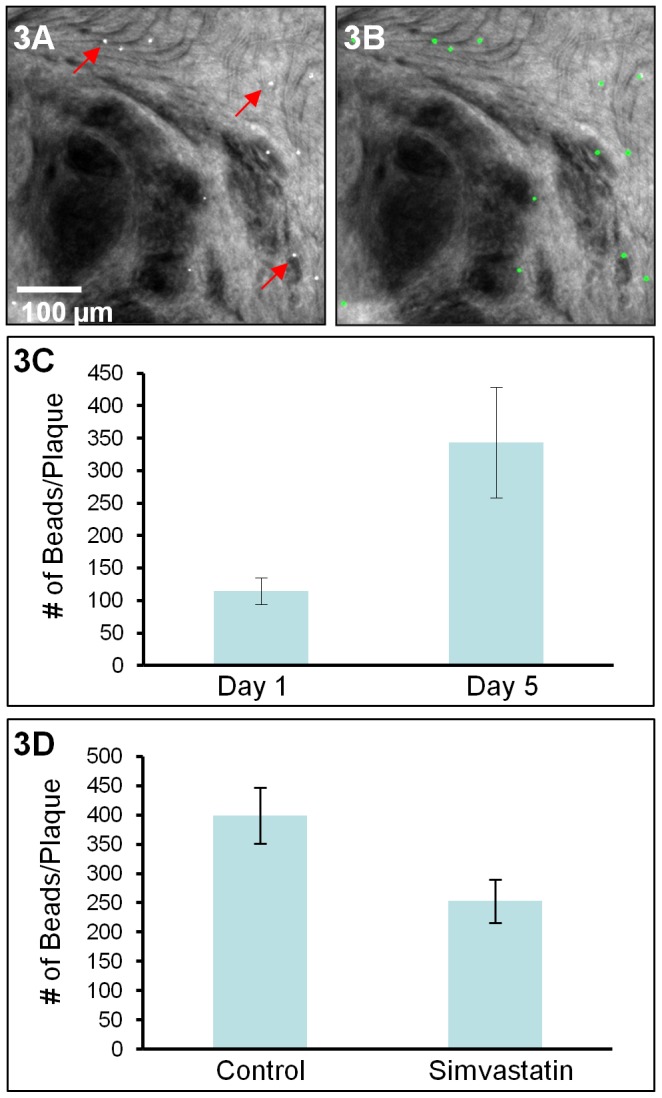
Quantification of bead-labeled monocyte-derived cells in atherosclerotic plaques. A) A sum projection of a z-stack collected from the lesser curvature of a murine aorta 1 day following subpopulation labeling. Examples of bead positive monocyte-derived cells are indicated by arrows. Automated bead counting was performed on a sum projection image generated from each z-stack in the composite multiphoton images. **B)** The result image shows segmentation of the beads identified by the algorithm (green circles). **C)** The number of bead-positive cells in each plaque at day 1 and day 5 following monocyte subpopulation labeling as determined by multiphoton microscopy. Five mice were examined per condition. Error bars ±SEM, p value = 0.02. **D)** The number of bead-positive cells in the plaques of ApoE^−/−^ mice treated with simvastatin or vehicle. Results show that simvastatin reduces non-classical monocyte recruitment to the plaque. Three mice were examined per condition. Error bars ±SEM, p value = 0.04.

Figure 2A–C shows examples of multiphoton composite images, collected at different wavelengths, of the entire atherosclerotic plaque of a murine aortic arch 1 day following labeling of monocytes with fluorescent beads. The composite images were generated by stitching sum projection images of 38 individual z-stacks. Each z-stack contains 23 images collected at 3 different wavelengths, so a total of 2622 images were used to generate the multiphoton composite images shown in Figure 2. The second harmonic generation image in Figure 2A shows widespread collagen at the surface of the plaque. On the same plaque, bead-positive monocytes are shown in green (Figure 2B). In Figure 2B, the bead diameter is dilated to 20 µm to facilitate visualization. Fitting with known patterns of monocyte accumulation, adherent bead-labeled monocytes show a specific distribution in the lesional regions, with a preferential accumulation at the edge of the plaque where shear stress is typically lower (red box Figure 2B) [31]. Areas rich in neutral lipids, such as cholesteryl esters and triglycerides, can be visualized via ORO fluorescence (arrows, Figure 2C). Figure 2D is an overlay of the composite images shown in A–C. Figure 2E, a white light image of the aorta shown in Figure 2A–D, is included for comparison. The tissue was stained with ORO to indicate the area for multiphoton imaging, so the plaque appears red. Specific bead-labeling of non-classical monocytes was confirmed by flow cytometry for both the day 1 and day 5 time points (Figure S1).

**Figure 4:**
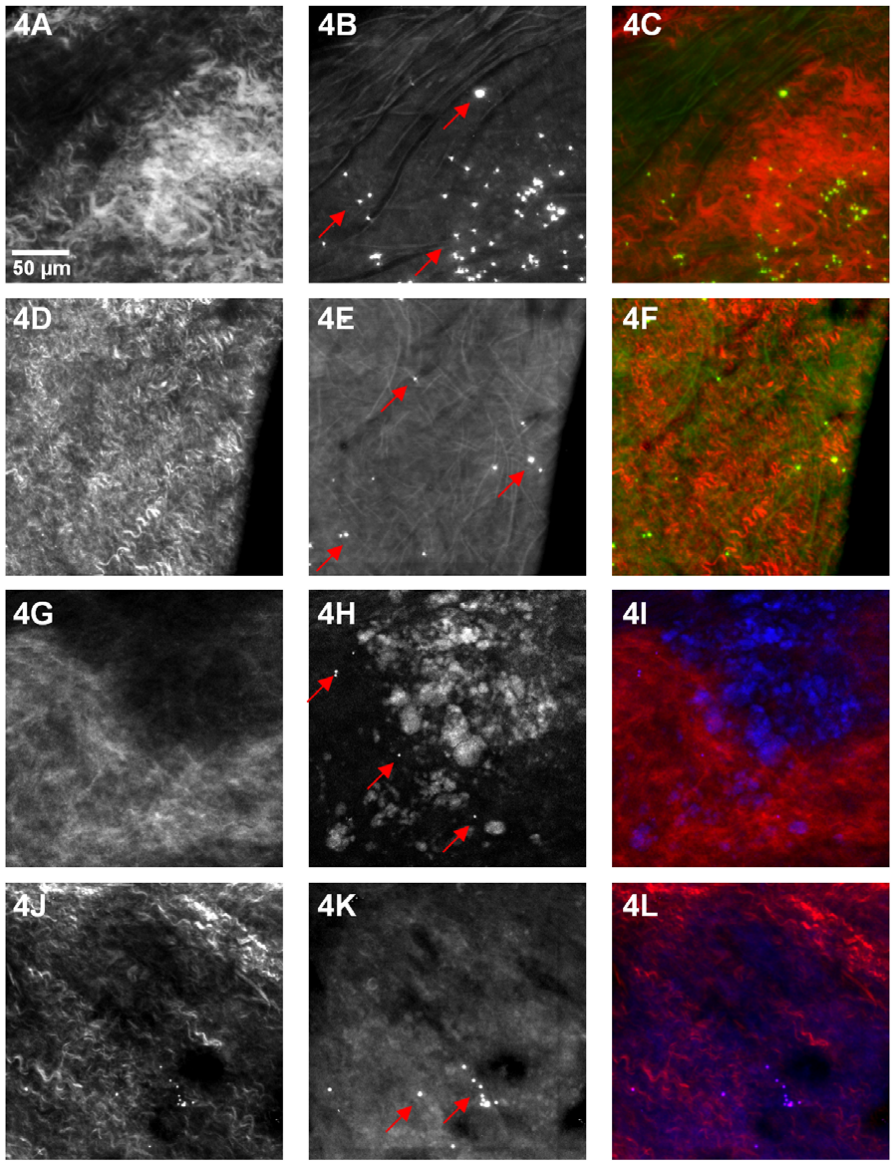
Visualization of plaque morphology and labeled monocytes. **A and G)** Second harmonic scattering from collagen in an ApoE^−/−^ control mouse treated with vehicle. **B)** Elastin and collagen autofluorescence in an ApoE^−/−^ control mouse. Several bead-positive monocytes, indicated by arrows, can be seen in regions containing collagen and elastin. **C)** Overlay. **D and J)** Second-harmonic scattering from collagen in an ApoE^−/−^ mouse treated with simvastatin. **E)** Elastin and collagen autofluorescence in an ApoE^−/−^ mouse treated with simvastatin. Several bead-positive monocytes, indicated by arrows, can be seen in regions containing collagen and elastin. **F)** Overlay. **H)** Neutral lipids, visualized as ORO fluorescence, in an ApoE^−/−^ control mouse. **K)** Neutral lipids, visualized as ORO fluorescence, in an ApoE^−/−^ mouse treated with simvastatin. Bead-positive monocyte-derived cells, indicated by arrows, are present in areas of lipid accumulation. Red (380 to 440 nm), green (490 to 530 nm) and blue (530 to 650 nm).

Automated bead counting was performed on a sum projection image generated from each individual z-stack collected with emission wavelengths 490 to 530 nm. Figure 3A displays a representative sum projection image of a single z-stack corresponding to an axial distance of 230 µm. Examples of bead-labeled monocytes or monocyte-derived cells are indicated by arrows. Figure 3B shows segmentation of the beads identified by the automated algorithm (green circles). In the image shown in Figure 3A, all beads were correctly identified. However, we note that clumps of beads occasionally present difficulty for the algorithm. In the day 1 vs. day 5 data, less than 5% of the beads are clumped. Despite areas for further algorithm refinement, results for the number of bead-positive cells in the plaque at day 1 and day 5 (Figure 3C) are consistent with those obtained via sectioning and manual counting in previously published data [6]. Both manual bead counting and multiphoton microscopy find 3 times more bead-positive Gr1^lo^ monocytes in the plaques on day 5 than on day 1.

**Figure 5:**
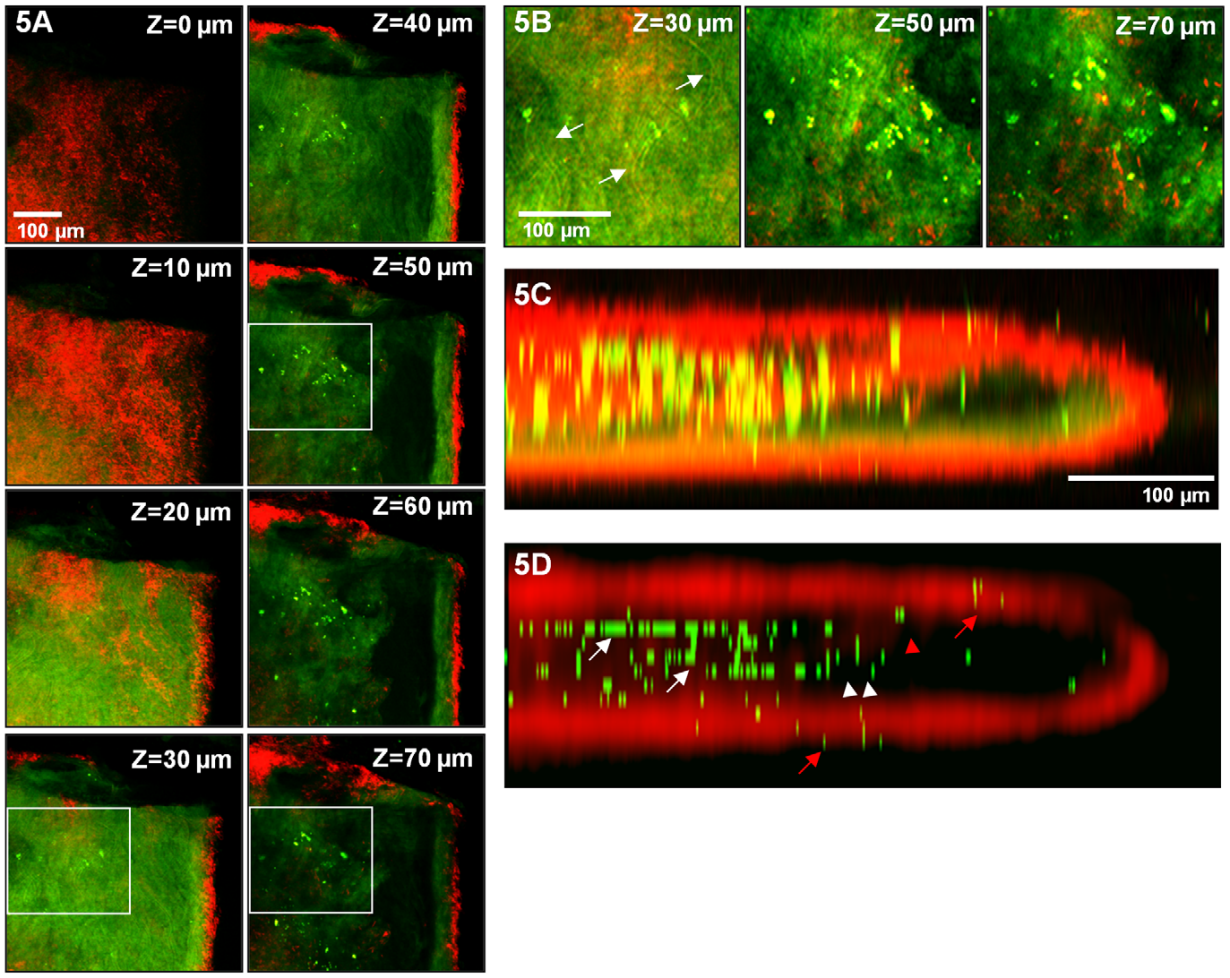
Multiphoton imaging of the brachiocephalic artery facilitates localization of bead-labeled cells to distinct plaque regions. Images were collected through the entire brachiocephalic artery of an ApoE^−/−^ mouse at depths of up to 200 µm. **A)** A series of xy planes acquired at different depths. The tunica media is shown in red. **B)** Enlarged views at different depths, highlighted by boxes in A. At a depth of 30 µm below the vessel surface, elastin fibers, presumably comprising the internal elastic lamina, are indicated by arrows. Images at greater depths are acquired from the atherosclerotic plaque, and bead-labeled monocyte-derived cells can be seen. **C)** A three-dimensional reconstruction of the brachiocephalic artery. The artery was mounted on a glass slide with a coverslip and thus appears compressed. Significant axial distortions, consistent with the optical inhomogeneity of tissue and the instrumentation employed, can be seen. To correct for the axial broadening of fluorescence in our data, beads were identified using the automated algorithm, and their signal was replaced by a bead image at the point of brightest fluorescence along the axial vector. **D)** A three dimensional reconstruction of the brachiocephalic artery in which beads identified in this manner replace the signal collected from 490 to 530 nm and the red channel is convolved with a 7×7-pixel Gaussian filter. Bead-positive cells can be seen in the plaque (white arrows), fibrous cap (white arrow heads) and shoulder (red arrow head) of the atherosclerotic plaque. Bead-positive cells can also be seen outside of the plaque (red arrows). Red (380 to 440 nm), green (490 to 530 nm).

We applied the same approach to investigate the effects of simvastatin on non-classical monocyte accumulation in plaques. It has been shown previously that simvastatin is a modulator of monocyte trafficking [30], [32]. Consistent with previous observations, we observed a 37%±9% reduction in Gr1^lo^ bead-positive monocytes in the plaques of mice treated with simvastatin compared to animals treated with vehicle (Figure 3D), indicating that simvastatin alters non-classical monocyte net accumulation.

These data demonstrate that multiphoton microscopy can accurately quantify the number of beads in plaques, thereby providing information on monocyte subpopulation trafficking while circumventing the need for mechanical tissue sectioning and manual bead counting. Further, data collection and analysis were largely automated, significantly reducing the amount of time necessary for quantification.

**Figure 6:**
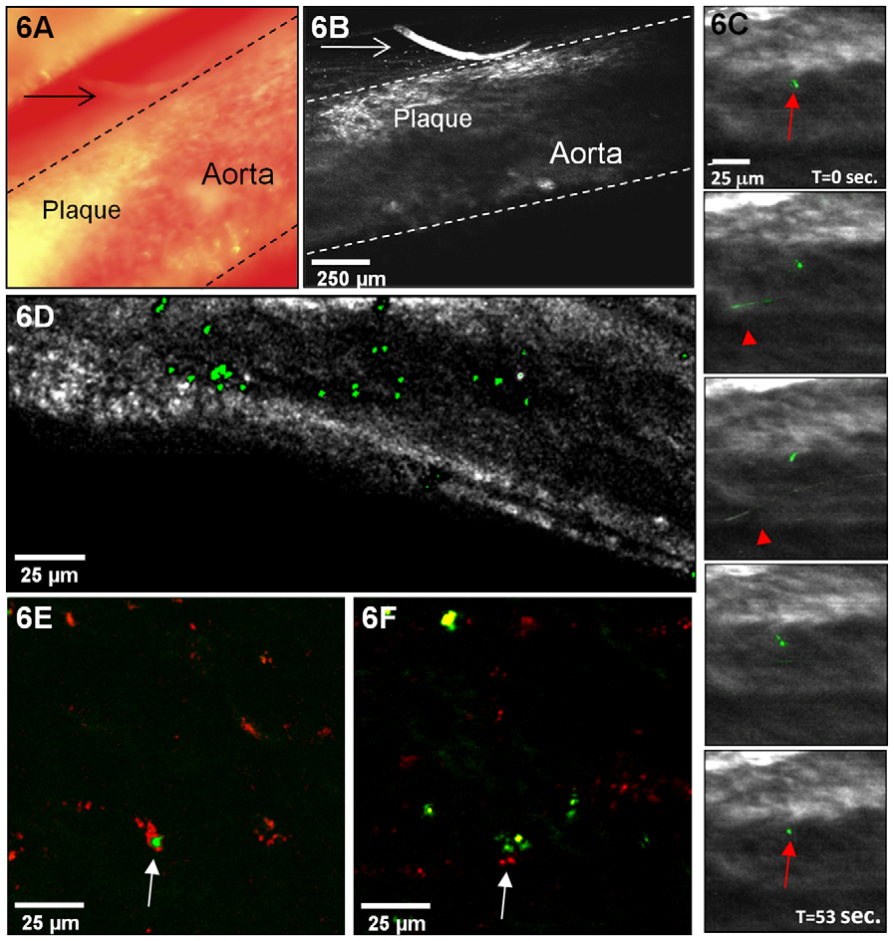
Intravital multiphoton microscopy allows observation of bead-labeled monocytes within murine atherosclerotic plaques. **A)** A white light image of an atherosclerotic plaque in the abdominal aorta of an ApoE^−/−^ mouse. **B)** A multiphoton image of the same region with excitation at 800 nm, collected from 490 to 530 nm. Dashed lines in each image demarcate the aorta. A hair which lies to the right of the atherosclerotic plaque (indicated by arrows in both A and B) is useful for orientation. **C)** Intravital images of bead-positive monocytes (green) as a function of time. Bead-positive monocytes that are circulating through the aorta may appear as lines in the image when their flow is synchronized with the raster scan of the microscope (examples are indicated by arrowheads). A stationary bead-positive monocyte was seen interacting with the endothelium of the atherosclerotic plaque (indicated by arrows). **D)** An intravital image demonstrating bead-positive monocyte-derived cell accumulation in an atherosclerotic plaque in the abdominal aorta of an ApoE^−/−^ mouse. **E and F)** Intravital images (excitation at 850 nm) of bead-positive monocyte-derived cells (green) interacting with fluorescently labeled low density lipoprotein (red) in an atherosclerotic plaque.

### Multiphoton Microscopy Reveals Monocyte Subpopulations within the Structural Components of the Plaque

In addition to providing information on monocyte trafficking, multiphoton microscopy allows tissue architecture to be visualized without the use of exogenous dyes. This time and effort saving advantage makes the approach convenient for determining the structural characteristics of atherosclerotic plaques [14]. In our data, monocytes, collagen, elastin and neutral lipids can be visualized in three dimensions. These data can be used to provide information on disease progression and to localize monocytes into different anatomical regions of the plaque (*e.g.*, necrotic core, shoulder, fibrous cap, or outside of the plaque in the adventitia).

Figure 4 displays representative images of the tissue architecture that can be visualized in our multiphoton data. Figure 4A, D, G and J show second-harmonic scattering from collagen. The images shown in Figure 4A–C and G–I are from an ApoE^−/−^ control mouse treated with vehicle, while those displayed in D–F and J–L are from an ApoE^−/−^ animal treated with simvastatin. Figure 4B and E shows elastin and collagen autofluorescence in the vessel wall of the aorta. Several bead-positive monocytes, indicated by arrows in 4B and E, can be seen in regions containing collagen and elastin. Accumulation of neutral lipids (cholesteryl esters) in macrophages and the extracellular space is a salient feature of atherosclerosis. In our samples, ORO was used to visually indentify regions for data acquisition, so neutral lipids can be seen as ORO fluorescence (Figure 4H and K). Bead-labeled monocyte-derived cells, indicated by arrows, are present in areas of lipid accumulation. Stronger ORO signals can be seen in 4H, the control mouse, than in 4K, the simvastatin treated animal. This is consistent with the cholesterol-lowering effects of simvastatin. We note that there have been reports of both autofluorescence and third-harmonic generation signals from neutral lipid deposits under multiphoton excitation [14], [33]. Thus, use of an exogenous fluorophore for neutral lipid visualization is not necessary. (ORO was used in this study to visually define the area for multiphoton data acquisition).

As an additional example of the tissue architecture that can be visualized using multiphoton microscopy, Figure 5 shows a three dimensional reconstruction of a brachiocephalic artery. Images were collected through the entire brachiocephalic artery at depths of up to 200 µm indicating that multiphoton microscopy is capable of imaging plaque through the vessel wall. The artery was mounted on a glass slide with a coverslip and thus may have been somewhat compressed. Figure 5A shows a series of xy planes acquired at different depths. The tunica media is shown in red. The contrast in the red channel is the result of second-harmonic generation. The adventitia is not clearly visible as it is dislodged during the aortic isolation and staining process. At a depth of 30 µm below the vessel surface, autofluorescent elastin fibers, presumably comprising the internal elastic lamina, are visible (Figure 5B). We note that the internal elastic lamina appears somewhat sheet-like (Figure 5A, z = 30 µm) because the images are acquired en face. Images at greater depths are acquired from the atherosclerotic plaque, and bead-positive monocyte-derived cells can be seen.

Due to the large area of data collection necessary to sample the entire atherosclerotic plaque (needed for accurate bead quantification), we employed a low magnification objective (10x). The low NA of this objective and the optical inhomogeneity of biological tissues compromise resolution. Thus, significant axial broadening can be seen in the three dimensional reconstruction shown in Figure 5C. We note that for applications that require superior resolution, higher NA objectives could be employed. To correct for the axial distortions in our data, beads were identified using an automated algorithm. Figure 5D shows a three dimensional reconstruction of the brachiocephalic artery in which the positions of beads identified in this manner replace the signal collected from 490 to 530 nm, and the red channel is convolved with a 7×7-pixel Gaussian filter. With this image processing, it is possible to differentiate beads in distinct anatomical regions of the plaque and artery. The fibrous cap of the plaque was identified via second-harmonic generation signals (red, collagen scattering) emanating from the lumen of the vessel. Bead-labeled cells (green) can be seen in the plaque (white arrows), fibrous cap (white arrow heads) and shoulder (red arrow head) of the atherosclerotic plaque. Bead-labeled cells can also be seen outside of the plaque (red arrows). The data demonstrate that multiphoton microscopy is able to image through the vessel wall to reveal the structural features of the atherosclerotic lesion at the luminal side of the vessel and differentiate beads in distinct anatomical regions of the plaque.

### Multiphoton Microscopy Allows Intravital Imaging of Monocyte Recruitment to the Atherosclerotic Plaque

The synergy of multiphoton microscopy and monocyte subpopulation bead-labeling allows intravital examination of monocyte trafficking and behavior, a realm not attainable with conventional methodologies. We employed time-lapse intravital multiphoton microscopy in mouse models of atherosclerosis to observe bead-labeled monocytes within the plaque. Figure 6A shows a white light image of an atherosclerotic plaque in the abdominal aorta of an ApoE^−/−^ mouse. A multiphoton image of the same region, collected from 490 to 530 nm with 800 nm excitation light, is shown in Figure 6B. Dashed lines in each image demarcate the aorta. A hair which lies to the right of the atherosclerotic plaque (indicated by arrows in both images) is useful for orientation. Column 6C shows intravital images of non-classical monocytes as a function of time (the entire time-lapse data set can be seen in Movie S1). Bead-labeled monocytes that are circulating through the aorta often appeared as lines in the image when their flow was synchronized with the raster scan of the microscope (examples are indicated by arrowheads). A stationary non-classical monocyte was seen interacting with the endothelium of the atherosclerotic plaque (indicated by arrows). This monocyte was present on the endothelium for the entire data collection session, ∼30 min, suggesting that visualization of diapedesis is possible with the approach. Figure 6C shows images during 53 sec. of the imaging period.

Figure 6D shows an image demonstrating the accumulation of bead-positive monocyte-derived cells, shown in green, in an atherosclerotic plaque in the abdominal aorta of an ApoE^−/−^ mouse. Injection of LDL fluorescently labeled with Alexa546 allows monitoring of the interaction of bead-positive monocytes or monocyte-derived cells and lipoproteins. Examples are shown in Figures 6E and F. The punctuate localization of LDL in Figures 6E and F (indicated by arrows) suggests that it has been internalized. These data provide direct *in vivo* visualization of monocyte trafficking and behavior in atherosclerotic plaques in living animals. The data shown demonstrate that monocyte accumulation in the plaque and foam cell formation can be visualized in real time in living animals with multiphoton microscopy.

## Discussion

We present a novel approach for the *in vivo* examination of monocyte subpopulations in mouse models of atherosclerosis. The combination of monocyte subpopulation bead-labeling and multiphoton microscopy results in an improved method for quantifying bead-labeled cells in excised tissues. This method is superior to conventional approaches in that it offers three dimensional high-resolution visualization of atherosclerotic lesions and bead-positive cell quantification in a manner that is largely automated. This was achieved through the use of optical rather than mechanical tissue sectioning and automated data collection and analysis. The image analysis tools used to quantify beads in the plaque provide rapid and objective alternatives to manual counting of bead-positive monocyte- derived cells. The data demonstrate accurate quantification of monocyte subpopulation trafficking in excised atherosclerotic plaques.

For the purposes of this study, we chose to focus on Gr1^lo^ monoctyes, since labeling them is more straightforward. Although we did not acquire data from Gr1^hi^ monocytes, we expect application of the approach to the examination of the Gr1^hi^ monocyte subpopulation to be identical to the examination of Gr1^lo^ monocytes. Gr1^lo^ but not Gr1^hi^ monocytes are stably labeled by intravenous injection of fluorescent beads, while Gr1^hi^ monocytes are specifically labeled when the fluorescent beads are injected after systemic depletion of blood monocytes and spleen macrophages [8]. As both labeling approaches use identical fluorescent beads, the application of multiphoton microscopy to their examination is expected to be identical.

The methodology also allows study of the interaction between vessel wall components and monocyte subpopulations without the need for tissue processing. Although, monocytes are known to enter the shoulder areas of the plaque (as can be seen in Figure 2B), little is known regarding the subsequent trafficking pattern of each monocyte subpopulation. The information provided by multiphoton microscopy could be used to examine the route of migratory egress of monocyte-derived cells from the plaque as well as provide information on disease progression.

Most exciting is the ability to extend the technique to the *in vivo* examination of monocyte trafficking, a realm not attainable with conventional methodologies. *In vivo* microscopic images with submicron resolution are highly desirable as immune processes are dynamic and thus are best studied in real time in living animals. In the end, the most meaningful behaviors of cells will be observed *in vivo*. Unlike the image of the brachiocephalic artery, shown in Figure 3, *in vivo* data must contend with motional artifacts, blood, which scatters both the incident and emitted signals and absorbs emitted light, and adipose tissue, which produces strong signals under multiphoton excitation. Thus, to date no study has shown monocyte recruitment to or trafficking in the plaque by intravital multiphoton imaging. As shown in Figure 6, we acquired intravital time-lapse multiphoton images of a non-classical monocyte entering an atherosclerotic plaque. We also imaged non-classical monocyte accumulation in the plaque and interaction with fluorescently labeled LDL. These data provide direct *in vivo* visualization of monocyte trafficking and behavior in atherosclerotic plaques in living animals. The data show that monocyte accumulation in the plaque and foam cell formation can be visualized in real time with intravital multiphoton microscopy.

Overall, much remains to be studied regarding the fate of monocyte-derived cell subpopulations in atherosclerosis. The data presented demonstrate that multiphoton microscopy has the potential to aid in and advance our understanding of the role of monocyte subpopulations in atherosclerosis. In addition, the ability to target and image monocyte subpopulations allows evaluation of therapies designed to selectively inhibit monocyte subpopulation recruitment or function. Results obtained using multiphoton microscopy will provide new insights into the role of monocyte subpopulations in atherogenesis and might eventually lead to innovative methods of promoting lesion regression. Further, the approach can be extended to study monocyte trafficking in virtually any organ system.

## Supporting Information

Figure S1 **Specific labeling of circulating non-classical monocytes** ***in vivo*** **. A**–**B)** Flow cytometry analysis following intravenous injection of latex beads. Blood was taken from mice 1 day (A) and 5 days (B) after bead injection. Representative plots are gated on all monocytes (CD115+, as in Figure 1A–C) and show specific labeling of Gr1^lo^ monocytes at both time points and very little labeling of Gr1^hi^ monocytes.
(TIF)

Movie S1 **Intravital time-lapse images of a bead-positive monocyte interacting with the endothelium of an atherosclerotic plaque.** A stationary bead-positive monocyte (green) was seen interacting with the endothelium of an atherosclerotic plaque. Bead-positive monocytes that are circulating though the aorta may appear as lines when their flow is synchronized with the raster scan of the microscope. Artifacts due to motions of the mouse, such as respiration, can be seen. Data were acquired with 800 nm excitation light. Emission was collected from 490 to 530 nm for 53 sec.
(AVI)
