# Supplementary figures and images for: Quantitative Analysis of Monocyte Subpopulations in Murine Atherosclerotic Plaques by Multiphoton Microscopy
Source: PLoS One. 2012 Sep 14;7(9):e44823. doi: 10.1371/journal.pone.0044823 (PMC3443108; doi:10.1371/journal.pone.0044823)

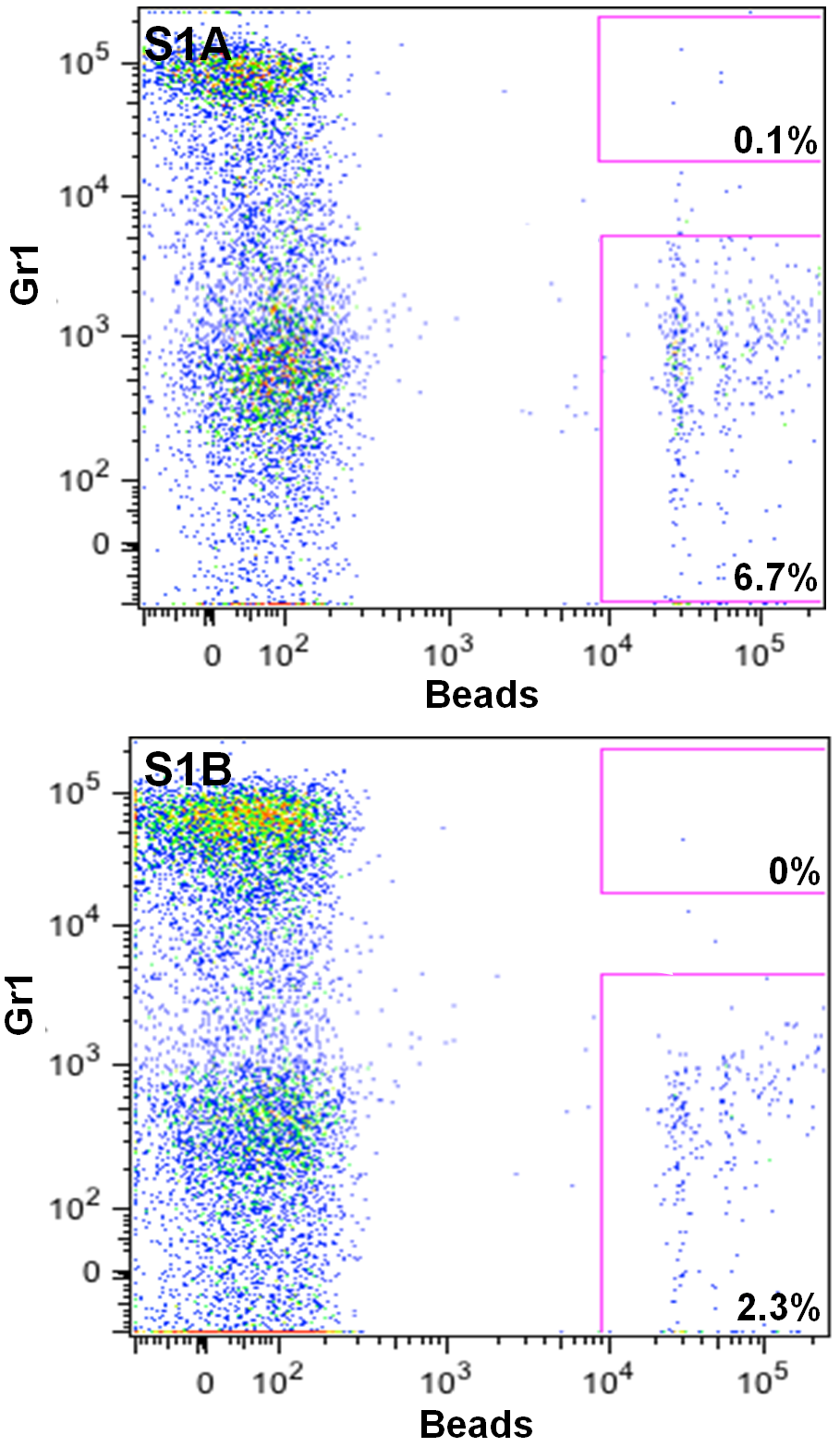

Supplement: Figure S1 — Specific labeling of circulating non-classical monocytes in vivo . A–B) Flow cytometry analysis following intravenous injection of latex beads. Blood was taken from mice 1 day (A) and 5 days (B) after bead injection. Representative plots are gated on all monocytes (CD115+, as in Figure 1A–C) and show specific labeling of Gr1lo monocytes at both time points and very little labeling of Gr1hi monocytes. (TIF) [file pone.0044823.s001.tif]
